# Psychological distress and anxiety compared amongst dental patients- results of a cross-sectional study in 1549 adults

**DOI:** 10.1186/s12903-019-0719-3

**Published:** 2019-01-31

**Authors:** Alexander Zinke, Christian Hannig, Hendrik Berth

**Affiliations:** 1 Technische Universität Dresden, Carl Gustav Carus Faculty of Medicine, Division of Psychological and Social Medicine and Developmental Neurosciences, Research Group Applied Medical Psychology and Medical Sociology, Fetscherstr. 74, 01307 Dresden, Germany; 2 Universitätsklinikum Carl Gustav Carus Dresden, Policlinic of Dental Maintenance, Fetscherstr. 74, 01307 Dresden, Germany

**Keywords:** Dental anxiety, Stress, Psychological, Depression, Adult, Dental clinic, Cross-sectional studies, Surveys and Questionnaires, Male, Female

## Abstract

**Background:**

This study aimed to identify anxiety in dental patients visiting a dental clinic using the Dental Anxiety Scale, their level of psychological distress using the Brief Symptom Inventory-18 and therefore identifying a correlation between these groups as well as their gender and age.

**Methods:**

An adult sample of *N* = 1549 patients (865 females, 779 males) was examined over the course of three years using the Brief Symptom Inventory-18 to evaluate psychological distress and the Dental Anxiety Scale to determine anxiety before dental treatment. Evaluations were conducted according to age and gender.

**Results:**

There was no correlation between different age groups of the sample the Dental Anxiety Scale. Anxiety, depression and GSI were more frequent in patients below the age of 46 than above. Women were more susceptible to signs of Anxiety and Somatization and scored higher on the Dental Anxiety Scale and the Global Severity Index than male patients. There was a significant positive correlation between scores of the BSI-18 categories: Somatization, Anxiety and Depression and the DAS for dental patients.

**Conclusions:**

This study showed that a relationship between dental anxiety and psychological distress exists. It would be an improvement to use a short questionnaire like the Dental Anxiety Scale to evaluate a patient before his first treatment so that more appropriate treatments can be pursued.

## Background

Dental fear and anxiety are not commonly directed towards the dental practitioner but more towards the treatment and the possibility of pain. Research by Weiner and Sheehan [1] further differentiated the origins into exogenic and endogenic. The influence can be environmental and due to stigmatizing experiences (exogenic) or part of generalized anxiety (endogenic). Patients with dental fear are a challenge for the dentist and the dental staff and can cause more stress responses [2]. Studies have shown that 70% of the general population feels anxious before a dental examination, 20% of that group are classified as highly anxious, and 5% evade dental treatments fully [3]. Dental anxiety, causing the patient to evade treatment, is a failure of modern dentistry to develop towards minimal invasiveness. Even though pain can be reduced to a minimum with modern day anesthetics, it seems that the fear of pain overshadows the actual pain. Anxiety disorder is pervasive, as 25% of all general practitioners can diagnose symptoms among their patients [4]. When questioned, patients label dental anxiety as the second biggest trigger for psychological distress at 21%, just after public speaking at 27%. It is essential for a dentist to define which group of patients is likely to have dental anxiety. A study conducted on 73 subjects with dental phobia showed that 40% of this sample had a current Axis I diagnosis other than a simple phobia, with an anxiety disorder (20%) being the most common [5]. Mental disorders such as anxiety disorder or depressive disorder could be highly associated with dental fear. Patients with post-traumatic stress disorder are affected most commonly.

Earlier studies exposed women to have a higher affinity for developing anxiety towards dental treatment [6]. Hakeberg compared the results of a male and female Swedish population with the Dental Anxiety Scale. Results showed a significantly higher dental anxiety in women between the age of 20 and 39 years compared to younger and older age groups [7]. A similar study by Stouthard found women between the ages of 26 to 35 to be most prone towards dental anxiety [8]. These results were confirmed in by Enkling.

Furthermore, it was determined that patients with a developed dental anxiety demand higher attention before, during and after the dental treatment [9]. Most patients wished for the most accurate information about the dental treatment they received, a sympathetic dentist and a pain-free dental examination. This evidence emphasised the significance of identifying dental fear in patients and being able to adapt towards their demands. Age seems to be a recurring factor, as fear of dental treatment is not common above the age of 60 [10–12]. Recent studies determine younger age groups to show higher levels of dental fear than older age groups [13]. However, it was also found that 15 to 25-year-olds showed fewer signs of dental fear than older individuals [14].

Due to these results, our aim in this study was to determine if there is a correlation between psychological distress measured with the frequently used BSI-18 and dental anxiety investigated by DAS. Furthermore, we tried to find a relationship between psychological distress and dental anxiety to age groups and gender.

## Methods

In different studies, throughout 3 years (2012–2015), approximately 2000 patients were asked to participate. Out of these, 1549 (77.5%) patients were included in our research. The data was collected independently in the research group for Medical Psychology and Medical Sociology in Dresden (Germany). DAS and BSI-18 questionnaires were handed out to dental patients before their routine treatments. Patients were also given a set of descriptive questions, collecting information about age and gender differences. The questionnaires were then examined in 2016 by our research group. All patients needed to be 18 years old and give written informed consent. Only patients providing written informed consent were included as study participants. All questionnaires were analyzed using IBM SPSS V23 to reveal significant similarities or differences. The mean total values were calculated and then analyzed using an independent sample t-test. Chi-squared tests were used to determine significance between questionnaire categories and sample characteristics. Cohens d respectively Cramers v were calculated as estimates of effect sizes. The coherence between dental anxiety and psychological distress were evaluated using Pearson correlations. *P* values equal or less than 0.05 were considered statistically significant. The research was conducted according to STROBE guidelines.

### Brief symptom Inventory-18

The Brief Symptom Inventory-18 (BSI-18) was first introduced in 2000 by Derogatis [15] as a further shortened BSI, which contained 53 items out of the first Symptom-Checklist 90-R. Developed as an instrument to define the state of psychological stress with only 18 items [16], the BSI-18 was used on cancer patients, victims of terrorist attacks, posttraumatic stress, alcohol addiction, and other cohorts. The three scales depression, anxiety, and somatization each contained six items and were combined to the Global Severity Index (GSI). The scores were between 0 and 90. Each of the 18 items is defined by a timeframe of the last seven days on a scale with four choices between “Not at all” and “Extremely.” The reliability of the three scales was assessed in 2010 on a sample of 638 psychotherapeutic patients: Somatization α = 12, Depression α = 0.84, Anxiety α = 0.84 and GSI α = 0.91 [17]. The reliability of the different BSI-18 categories in our study was α = 0.80. The BSI-18 determined the level of psychological distress in this study because patients were able to complete it quickly, its reliability and its quick overview of psychological symptoms.

### Dental anxiety scale

The Dental Anxiety Scale - DAS was first introduced in 1969 by Corah and is used widely to assess dental fear in patients [18, 19]. It consists of four questions based on situations before and during dental treatment. The patients self-assessed themselves. Each issue was scored from 1 to (low anxiety) to 5 (high anxiety). The possible range of scores was 4 to 20 and therefore allowed the patient to be categorized into proven dental anxiety if the score was above a cut-off value of 15 [20], some anxiety at a value between 13 and 15 and little to none anxiety below a score of 13. The reliability of the Dental Anxiety Scale was rtt = 0.86 [18]. In our study, the DAS had a reliability of α = 0.88. This questionnaire was used to determine the level of dental anxiety in this study due to its shortness, as well as its scientifically proven reliability.

## Results

In total, 1549 patients completed the questionnaires. Fifty-six percent of the patients were female (865/1549 patients), and 51 % of patients were at or above the age of 46 (779/1525). The mean age was 45.68 years (*SD* 18.70, range 18–88 years).

### Age compared to dental anxiety and psychological distress

Ninety-nine percent (1527/1549) of all patients were divided into those younger than 46 years old (748/1527) and those at or above the age of 46 (779/1527) to be able to compare the averages of “younger” as well as “older” patients. The age 46 years was chosen due to it being the average age of our sample group. This data was then compared to the DAS category, and the patient was placed according to their score on the questionnaire (Table 1).

**Table 1:** Age of patients compared to dental anxiety categories (DAS) and psychological distress (BSI-18)

		Age		
Dental anxiety	Total sample (*N*, %)	< 46 years (*N*, %)	≥ 46 years (*N*, %)	Chi2-test
Low anxiety	1072 (70)	517 (69)	555 (71)	χ2 (2, *N* = 1527) = 0.95, *p* = 0.62, v = 0.457
Moderate anxiety	379 (25)	191 (26)	188 (24)	
High anxiety	76 (5)	40 (5)	36 (5)	
Psychological distress	Total sample (*M*, SD)	< 46 years (*M*, SD)	≥ 46 years (*M*, SD)	t-test
Somatization	1.94 (2.90)	1.93 (2.76)	1.95 (3.03)	*t*(1425) = −0.16, *p* = 0.874), d = − 0.008
Depression	1.88 (3.35)	2.33 (3.45)	1.43 (3.17)	*t*(1423) = 5.16, *p <* 0.0001, d = 0.274
Anxiety	2.78 (3.53)	3.20 (3.31)	2.39 (3.70)	*t*(1425) = 4.31, *p <* 0.0001, d = 0.228
GSI	6.59 (8.21)	7.44 (7.91)	5.76 (8.38)	*t*(1426) = 3.89, *p <* 0.0001, d = 0.206

Most patients of both age groups were placed in the low anxiety category (1072/1528). In all categories, the spread of the two age groups was similar. There were more patients below the age of 46 in the high anxiety category than those at or above the age of 46. There were slightly more patients at or above the age of 46 in the low anxiety age group than those below the age of 46. There was no statistical significance found.

The same age groups were then divided into the BSI-18 categories Somatization, Depression and Anxiety, as well as the Global Severity Index (Table 1). Patients younger than 46 years old had approximately the same score as patients at or above 46 in the category Somatization. In the category Depression, younger patients had a higher average than older patients. This proved to be statistically significant. Anxiety was higher in younger patients than older patients. A significance was found. The overall Global Severity Index was higher for younger patients than older patients.

### Gender compared to dental anxiety and psychological distress

The Dental Anxiety Scale was broken down into its categories and then searched for significant differences between female and male patients (Table 2). Most patients are classified as having low dental anxiety (1084/1544). Patients with a high anxiety of dental treatment were in the minority. A statistically significant difference was found.

**Table 2:** Gender of patients compared to dental anxiety categories (DAS) and psychological distress (BSI-18)

		Sex		
Dental anxiety	Total sample (*N*, %)	Male (*N*, %)	Female (*N*, %)	Chi2-test
Low anxiety	1084 (70)	510 (75)	574 (67)	χ2 (2, *N* = 1544) = 14.97, *p* = 0.001, v = 0.98
Moderate anxiety	384 (25)	153 (22)	231 (27)	
High anxiety	76 (5)	21 (3)	55 (6)	
Psychological distress	Total sample (*M*, SD)	Male (*M*, SD)	Female (*M*, SD)	t-test
Somatization	1.94 (2.90)	1.73 (2.69)	2.10 (3.04)	*t*(1418) = −2.47, *p* = 0.014, d = −0.131
Depression	1.88 (3.35)	1.68 (3.47)	2.03 (3.24)	*t*(1437) = −1.94, *p* = 0.052, d = − 0.102
Anxiety	2.78 (3.53)	2.29 (3.07)	3.19 (3.80)	*t*(1438) = −4.96, *p <* 0.0001, d = − 0.262
GSI	6.59 (8.21)	5.69 (7.97)	7.31 (8.29)	*t*(1384) = −3.76, *p <* 0.0001, d = − 0.202

Table 2 shows the average scores achieved by female and male patients. Female patients had higher scores in the BSI-18 categories Somatization, Depression, Anxiety and the Global Severity Index. Results given by the DAS score can also be defined as statistically significant. Female patients achieved higher scores than male patients.

### Comparing the relation between psychological distress (BSI-18) and dental anxiety (DAS)

A Pearson correlation was completed to compare the outcomes of the BSI-18 questionnaire and the DAS (Table 3). The results showed significant positive correlations between all BSI-18 categories and the DAS questionnaire.

**Table 3:** Correlation between dental anxiety (DAS) and psychological distress (BSI-18)

	DAS		
	Pearson Correlation	Sig. (2-tailed)	*N*
Somatization	0.257	*p <* 0.0001	1439
Depression	0.158	*p <* 0.0001	1437
Anxiety	0.337	*p <* 0.0001	1439
GSI	0.301	*p <* 0.0001	1440

## Discussion

Comparing the categories of the Dental Anxiety Scale to patients at or above and below the age of 46 showed no statistical significance. These findings were strongly in contrast to those of similar studies [10, 12, 21–24] showing a decrease in the prevalence of dental anxiety in older populations compared to younger populations. An explanation could be the classification into only two age groups and a generalization of the words “young” and “old”. A flaw of the Dental Anxiety Scale is the non-inclusion of local anesthetics and a strong influence of a patients judgment of the treatment. Patients are most afraid of the pain and bodily harm done by the injection [25], even though 40% of younger age groups prefer a treatment with anesthesia [26]. The Modified Dental Anxiety Scale (MDAS), modeled on the original anxiety scale by Corah, includes an additional question about anesthetics [10]. Findings for the sample groups showed a connection between age groups and dental anxiety using the MDAS [21].

The Brief Symptom Inventory-18 was used to compare the same age groups. Findings indicated a higher presence of depression and anxiety in younger than in older patients. Increased experience, emotional control, and immunity to stressful experiences are possible factors supporting these results [27]. The older patient might be not working anymore and therefore can recover and prepare better for stressful experiences. There are, however, indications that symptoms of anxiety and depression begin to increase again [28]. Therefore, it is important not to assume an older patient to not be at risk for anxiety and depression symptoms. These results further emphasize the need for a comfortable and reliable dental anxiety screening method.

When comparing dental anxiety and the Global Severity Index with the gender of our sample, female patients were more susceptible to psychological distress regarding a dental treatment than male patients. This was a confirmation of earlier studies [4]. Women have a higher chance of developing dental anxiety than male patients as we expected [29]. An explanation for this might be higher levels of neuroticism in women than men and it being correlated to anxiety [30–32].

Correlations between psychological distress and dental anxiety might be an indicator that patients with a generally higher level of psychological symptoms (depression, somatisation, anxiety) are at a higher risk for the development of specific anxieties such as dental fear. A similar study, comparing 212 patients with psychosomatic service with 95 healthy controls, confirmed these results [33]. The large sample size of this study was favorable in comparison to similar studies. Also, the use of a general population and not just patients with an existing diagnosis of anxiety allowed a non-biased view towards the results. However only patients voluntarily visiting a dentist were interviewed. Patients with a diagnosable dental anxiety and an aversion to dental visits could not be examined.

The BSI-18 and the DAS are scientifically reliable questionnaires and are frequently used for large sample sizes, as in this study. To improve the individual judgment of the patient’s well-being, the more detailed BSI with 53 items or even the SCL-90R with 90 items could be applied in a subsequent study. There are also several factors for which the Dental Anxiety Scale is often criticised, such as the answering scheme not being consistent across the questionnaire and, most importantly, not including a question about local anaesthetics. The Modified Dental Anxiety Scale, validated by Humphris, Morrison and Lindsay in 1995 [34], improved these flaws and can be an interesting alternative to Corah’s DAS.

All questionnaires were completed by the judgment of the patients themselves. It is therefore possible that some patients did not answer the questions truthfully and might have reduced the severity of their answers to avoid being singled out as a patient with dental anxiety. The treatment the patients were expecting after their survey was not captured on any questionnaire. Patients with acute pain might already be psychologically weakened, expecting more pain and therefore fearing the treatment more than someone waiting for a routine dental check-up.

Patients suffering from dental anxiety are restricted in their daily routine. Most of the time, these patients will only choose to make a dental appointment if the pain becomes too unbearable. If the dentist is unaware of the patient’s anxiety, the encounter can deteriorate. This should not be the experience first experience of an individual fearing dental treatment. Screening using the Dental Anxiety Scale is fast and easy and can prepare the dentist to handle the patient. This would, however, require that the dentist has been professionally prepared to treat a patient who fears his surroundings. Improvements can be made in preparing students during their training or given as a mandatory lecture. With the help of specialized practitioners and a fully prepared dentist, it may be possible to reduce dental anxiety in susceptible individuals.

## Conclusions

Using a large sample of dental patients the results of the study showed strong associations between dental anxiety and psychological distress (somatization, depression, anxiety). Patients below the age of 46 and women report more psychological distress. Additionally, women described more dental anxiety compared to male patients.

## Abbreviations

BSI-18: Brief Symptom Inventory-18
DAS: Dental Anxiety Scale
GSI: Global Severity Index, total Score of the Brief Symptom Inventory-18
IBM SPSS: Statistical Software for the Social Sciences
MDAS: Modified Dental Anxiety Scale
SCL-90R: Symptom-Checklist-90 Revised
STROBE: Strengthening the Reporting of Observational studies in Epidemiology
